# Nitrogen addition level is more influential to aboveground productivity than nitrogen addition frequency in an alpine grassland

**DOI:** 10.3389/fpls.2026.1821183

**Published:** 2026-08-14

**Authors:** Junjie Liu, Chao Liu, Wanqing Zhao

**Affiliations:** 1 College of Ecology and Environment, Xinjiang University, Urumqi, China; 2 Technology Innovation Center for Ecological Monitoring and Restoration of Desert-Oasis, Ministry Nat Resources Desert, Urumqi, China; 3 Key Laboratory of Oasis Ecology, Ministry of Education, Xinjiang University, Urumqi, China

**Keywords:** aboveground productivity, Bayanbulak alpine grassland, nitrogen addition frequency, nitrogen addition level, plant functional groups, soil physicochemical properties

## Abstract

**Aims:**

The combined effects of climate change and human activities have greatly increased atmospheric nitrogen deposition globally, with profound impacts on plant growth and grassland productivity. This study investigates the mechanisms governing how plant aboveground productivity responds to nitrogen addition with varied addition levels and frequencies in Bayanbulak alpine grassland.

**Methods:**

A field experiment was conducted with five N addition levels (0, 5, 10, 15, 20 g·m^-2^) and two frequencies (low and high). Soil properties, plant aboveground biomass and nutrient content were analyzed using ANOVA, Mantel tests, and structural equation modeling (SEM).

**Results:**

Nitrogen addition level was the primary driver of changes in soil properties and plant productivity. Community aboveground net primary productivity (ANPP) increased significantly with nitrogen addition level (F = 10.930, P< 0.001). Poaceae ANPP increased significantly with nitrogen addition level (p = 0.009), whereas Rosaceae and Fabaceae showed no significant response but exhibited progressive phosphorus limitation under high nitrogen levels. Significant level-dependent increases were observed for soil nitrate nitrogen (soil NN) and available phosphorus (soil AP) (p< 0.05). Level × frequency interactions were significant for several soil variables, while addition frequency alone had limited main effects. SEM further revealed that the direct effect of nitrogen addition level on ANPP was significant for Poaceae under high-frequency addition, with additional indirect mediation via plant C:N. Nitrogen addition indirectly affected Rosaceae ANPP via plant TN, TP, C:P and N:P, with TP and C:P exerting negative effects; while for Fabaceae, no significant pathway detected, indicating its insensitivity to nitrogen enrichment. Plant nutrient content was a stronger predictor of ANPP than soil properties.

**Conclusions:**

Nitrogen addition level exerts a stronger and more direct influence on aboveground productivity than frequency, indicating that total nitrogen load, rather than its temporal pattern, is the key driver of productivity changes. Functional groups show decoupled N–P dynamics: Poaceae benefit from alleviated N limitation, while Rosaceae and Fabaceae suffer progressive P limitation, and suppressed N fixation in Fabaceae. Predicting N deposition impacts thus requires considering these functional-group-specific responses.

## 1 Introduction

Since the Industrial Revolution, climate change and human activities have substantially increased atmospheric nitrogen deposition worldwide (Galloway et al., 2008). At the beginning of the 20th century, global atmospheric nitrogen deposition stood at approximately 103 Tg·a^-1^, a figure projected to reach 200 Tg·a^−1^ by 2050 (Galloway et al., 2004). In China, the average deposition rate nearly doubled between 1980 and 2010, rising from 13.2 to 21.1 kg·N·hm^-2^·a^-1^ (Cao F. F. et al., 2021; Liu et al., 2013), making China the world’s third-largest nitrogen deposition region, after Europe and the United States.

Grassland ecosystems are among the most extensive and vital terrestrial ecosystems, playing a critical role in global carbon and nitrogen cycling as well as climate regulation (Lei et al., 2016; Liu et al., 2023). Given their importance, the impact of nitrogen deposition on grassland has become a central research focus in ecology (Li T.P. et al., 2019). Nitrogen deposition alters plant stoichiometry by disrupting internal carbon, nitrogen, and phosphorus balances, thereby influencing competitive and facilitative interactions among species and ultimately reshaping grassland ecosystem structure and function (Sun et al., 2018). Numerous nutrient addition experiments have been conducted worldwide to investigate the effects of nitrogen deposition. Increased nitrogen deposition can enhance soil nitrogen availability, promote plant growth and boost grassland productivity (Li et al., 2015, 2020). On the other hand, excessive nitrogen deposition may cause soil acidification, disrupt plant nutrient balance, and favor nitrophilic species, leading to reduced community diversity (Cao J. et al., 2021; Shen et al., 2022; Zhang R, 2022). Numerous studies have investigated the effects of nitrogen addition on aboveground net primary productivity (ANPP) and plant growth in grassland ecosystems, yet the results remain inconsistent due to variations in climate, grassland type, nitrogen addition level, application frequency, and experimental duration (Bai et al., 2010; Hu et al., 2022). These divergent outcomes may also arise from asynchronous responses among plant functional groups during competition for soil nitrogen. Coexisting plant species often differ in their nitrogen acquisition strategies. Recent studies have shown that species exhibit distinct preferences for different nitrogen forms—ammonium, nitrate, and amino acids—leading to chemical niche differentiation that reduces direct competition for soil nitrogen (Yang et al., 2022; Cao et al., 2021). Such species-specific nitrogen form preferences are consistent with resource-based niches, which posit that differentiation in the uptake of different nitrogen forms provides a basis for species diversity and dominance (McKane et al., 2002). Furthermore, different species exhibit distinct phenological strategies and rooting depths, allowing them to acquire nitrogen at different times and from different soil layers (Fargione and Tilman, 2005). Plant nitrogen uptake is also seasonally dynamic, with coexisting species exhibiting temporal niche differentiation in nitrogen form utilization (Gao et al., 2025). Collectively, these functional group-specific nitrogen acquisition strategies—varying in time, space, and chemical form—can generate asynchronous community-level responses to nitrogen enrichment, thereby contributing to the variability in ANPP responses observed across studies.

Soil physicochemical properties, such as moisture, pH, and salinity, play critical roles in regulating plant growth and nutrient availability (Deekshitha et al., 2021; Xie et al., 2015; Wang et al., 2021). Therefore, understanding the effects of both nitrogen addition level and frequency is essential for accurately predicting the ecological consequences of atmospheric nitrogen deposition in alpine grassland ecosystems. Previous studies have primarily focused on the effects of nitrogen addition levels on grassland productivity (Bai et al., 2010; Hu et al., 2022), while much less attention has been paid to the role of addition frequency. The frequency of nitrogen inputs can influence soil nitrogen availability dynamics, plant nutrient uptake strategies, and microbial activity, potentially leading to different ecological outcomes compared to a single large application event (Zhang et al., 2015; Wang et al., 2018). Recent studies have shown that nitrogen addition frequency can significantly alter soil chemical properties, including soil pH, base cations, and micronutrient availability (Wang et al., 2018; Li et al., 2019), which in turn may affect plant productivity. However, the total amount of nitrogen input is generally considered the primary determinant of plant productivity in nitrogen-limited ecosystems, whereas the temporal distribution of inputs plays a secondary role (Bai et al., 2010). A recent study in temperate grasslands further demonstrated that aboveground productivity depends more on the rate than on the frequency of nitrogen addition (Zhang et al., 2015).

The Bayanbulak alpine grassland, located in the Tianshan Mountains, is recognized as China’s largest subalpine alpine grassland and the country’s second-largest grassland (Hu et al., 2016, 2019). Given its unique geographical and climatic conditions, the conservation and sustainable management of this grassland are critical for regional ecosystem stability, socio-economic development, and desertification control in arid areas. In addition to the total amount of nitrogen deposition, the frequency of deposition events may also play a critical role in shaping ecosystem responses. In the Tianshan Mountains region, atmospheric nitrogen deposition is dominated by wet deposition, which delivers nitrogen to ecosystems through multiple, relatively low-intensity precipitation events during the growing season (Wang et al., 2012). Evidence from the Qilian Mountains, which share similar climatic and ecological characteristics with the Tianshan region as a transition zone between the Tibetan Plateau and arid areas, indicates that nitrogen deposition occurs primarily as wet deposition during precipitation events, with nitrate nitrogen accounting for a substantial proportion of dissolved inorganic nitrogen (Zhang B et al., 2022). This pattern is largely driven by regional climatic conditions, including seasonal precipitation distribution and atmospheric transport pathways.

Previous studies have consistently shown that different plant functional groups exhibit divergent responses to nitrogen enrichment. Graminoids (Poaceae) generally benefit from increased nitrogen availability due to their rapid growth rates, tall stature, and efficient nutrient acquisition strategies, which allow them to compete effectively for light and soil resources (Bai et al., 2010; Stevens et al., 2006). In contrast, forbs (including Rosaceae and other dicotyledonous species) often decline under nitrogen addition, partly because they are outcompeted by grasses for light and partly due to soil acidification and ammonium toxicity (Stevens et al., 2006; Zhang et al., 2014). Leguminous plants (Fabaceae) typically show weaker or negative responses to nitrogen addition, as elevated soil nitrogen availability suppresses symbiotic nitrogen fixation, reducing their competitive advantage over non-fixing species (Streeter and Wong, 1988; Van Kessel and Hartley, 2000). These contrasting responses among functional groups can drive shifts in community composition and alter ecosystem productivity under increasing nitrogen deposition. Foliar nutrient concentrations are sensitive indicators of plant responses to nitrogen addition, as they directly reflect changes in soil nutrient availability and plant internal nutrient dynamics (Mao et al., 2020). Therefore, plant nutrient traits may provide a more proximate and integrated measure of plant growth status than soil physicochemical properties alone, which are often spatially heterogeneous and influenced by multiple interacting factors.

How nitrogen addition level and frequency jointly affect plant aboveground productivity across different functional groups remains poorly understood, particularly in alpine grasslands of Central Asia.

To address this knowledge gap, we conducted a field experiment in the Bayanbulak alpine grassland to evaluate how nitrogen addition level and frequency influence ANPP of different plant functional groups, and to investigate the mediation roles of soil physicochemical properties, plant nutrient traits. Specifically, we aimed to answer: (1) how do ANPP and nutrient traits of different functional groups respond to nitrogen addition level and frequency; (2) how do nitrogen addition level and frequency influence soil physicochemical properties; (3) what are the coupling mechanisms among soil physicochemical properties, aboveground productivity and plant nutrient characteristics under different nitrogen addition regimes? While previous studies have largely focused on the effects of nitrogen addition rates on grassland productivity, few have simultaneously manipulated both the level and frequency of nitrogen inputs to assess their relative importance across different plant functional groups. Moreover, the mechanisms by which nitrogen addition frequency affects soil-plant nutrient coupling and functional group-specific productivity remain poorly understood, particularly in alpine grasslands. This study therefore provides novel insights by independently comparing the effects of nitrogen addition level versus frequency on ANPP, identifying functional group-specific responses (Poaceae vs. Rosaceae vs. Fabaceae), and exploring the potential pathways through which nitrogen addition might induce phosphorus limitation and suppress symbiotic nitrogen fixation to constrain the productivity of non-grass species, using a combination of randomized forest modeling and structural equation modeling.

Based on previous findings, we propose the following hypotheses: (1) Nitrogen addition level will have a stronger effect on soil properties and plant productivity than addition frequency. (2) Plant functional groups will exhibit divergent responses: Poaceae productivity will increase with nitrogen addition level, whereas Fabaceae productivity will show a weaker or negative response due to suppression of symbiotic nitrogen fixation. (3) Plant nutrient content will be a more direct predictor of aboveground productivity than soil physicochemical properties.

## 2 Materials and methods

### 2.1 Study area

**Figure 1:**
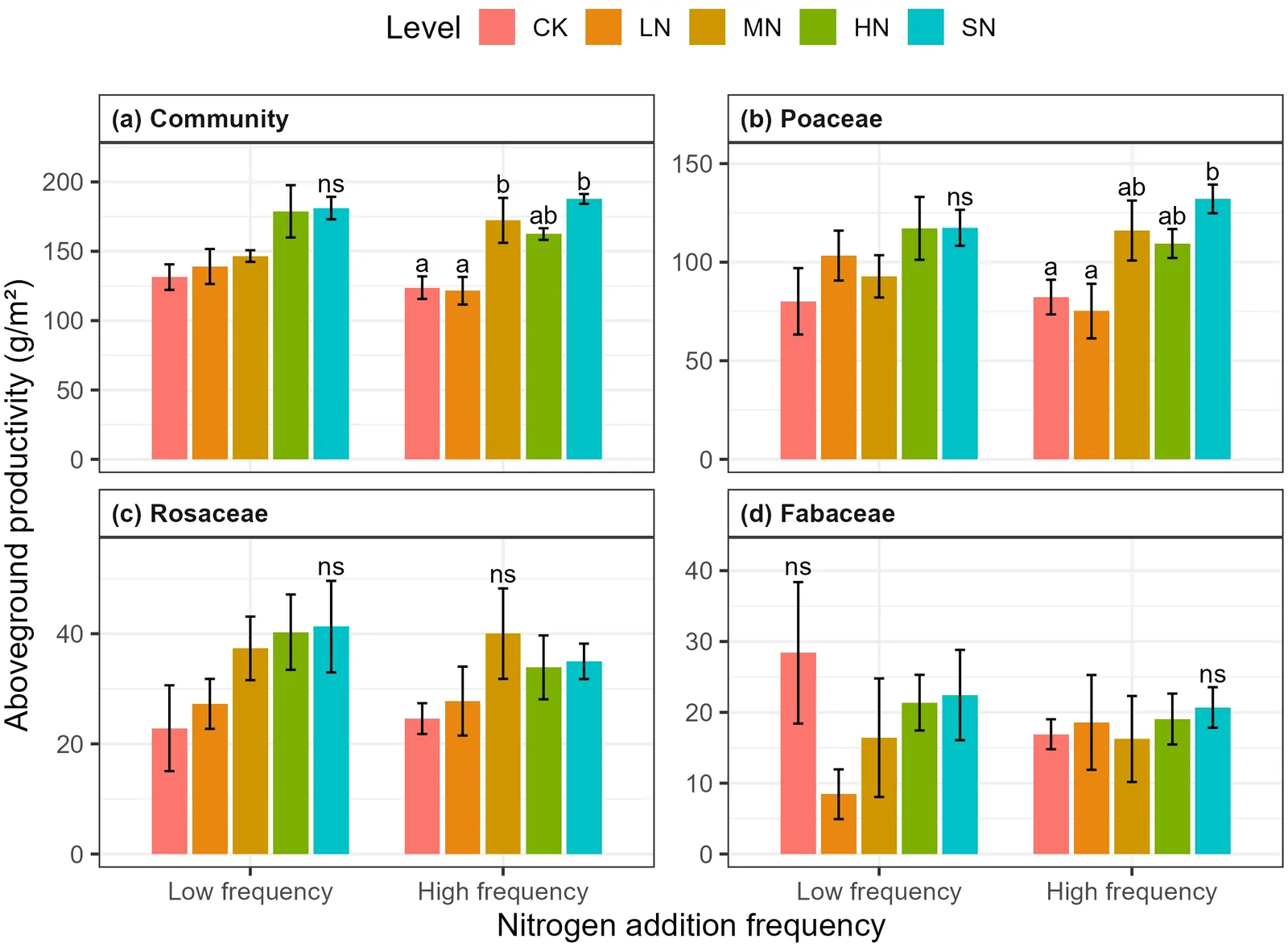
Effects of nitrogen addition level and frequency on aboveground biomass of **(a)** community, **(b)** Poaceae, **(c)** Rosaceae, and **(d)** Fabaceae. Bars represent means ± SE (n = 4). Different lowercase letters indicate significant differences among nitrogen addition levels within each frequency at p< 0.05 (one-way ANOVA, Tukey’s HSD). “ns” denotes no significant differences among levels within a given frequency. CK, control; LN, low nitrogen addition; MN, medium nitrogen addition; HN, high nitrogen addition; SN, severe nitrogen addition.

The study area is located near the Bayanbulak Grassland Ecosystem Research Station of the Chinese Academy of Sciences in Hejing County, Bayin’guoleng Mongol Autonomous Prefecture, Xinjiang, China (42°88’N, 83°70’E; see **Figure 1**). The region features a typical alpine climate, with an average elevation of approximately 2,470 m. Mean annual temperature is -4.6 °C, with extremes ranging from 25.4 °C to -40.5 °C. Annual precipitation averages about 270 mm, 60–80% of which falls during the growing season. The soil is classified as chestnut calcium soil, and the sampling site is topographically flat with homogeneous vegetation cover. Prior to the nitrogen addition experiments, the area had been enclosed for five years.

Dominant species included *Poa pratensis* L. and *Agropyron cristatum* (Linn.) Gaertn., *Koeleria cristata* (L.) Pers. and *Festuca ovina* L. The relative abundances of vegetation in study area are shown in **Table 1**, with Poaceae comprising approximately 66% of the total plant individuals, indicating its functional dominance in this alpine grassland, followed by Fabaceae (13.6%) and Rosaceae (8.0%). The remaining proportion consisted of other forbs.

**Table 1:** Dominant plant species and their relative abundances in the study site.

Family	Species	Proportion of total individuals
Poaceae	*Koeleria cristata* (L.) Pers.	13%
Poaceae	*Agropyron cristatum* (Linn.) Gaertn.	18%
Poaceae	*Festuca ovina* L.	13%
Poaceae	*Poa pratensis* L.	22%
Fabaceae	*Medicago sativa* L.	5.5%
Fabaceae	*Astragalus adsurgens* (Fisch.) Bunge.	5.5%
Fabaceae	*Oxytropis glabra* DC.	2.6%
Rosaceae	*Potentilla fragarioides* L.	5.4%
Rosaceae	*Potentill abifurca* Linn.	2.6%
Compositae	*Taraxacum mongolicum* Hand.	2.5%
Gentianaceae	*Gentiana macrophylla* Pall.	1.5%

### 2.2 Experimental design

**Figure 2:**
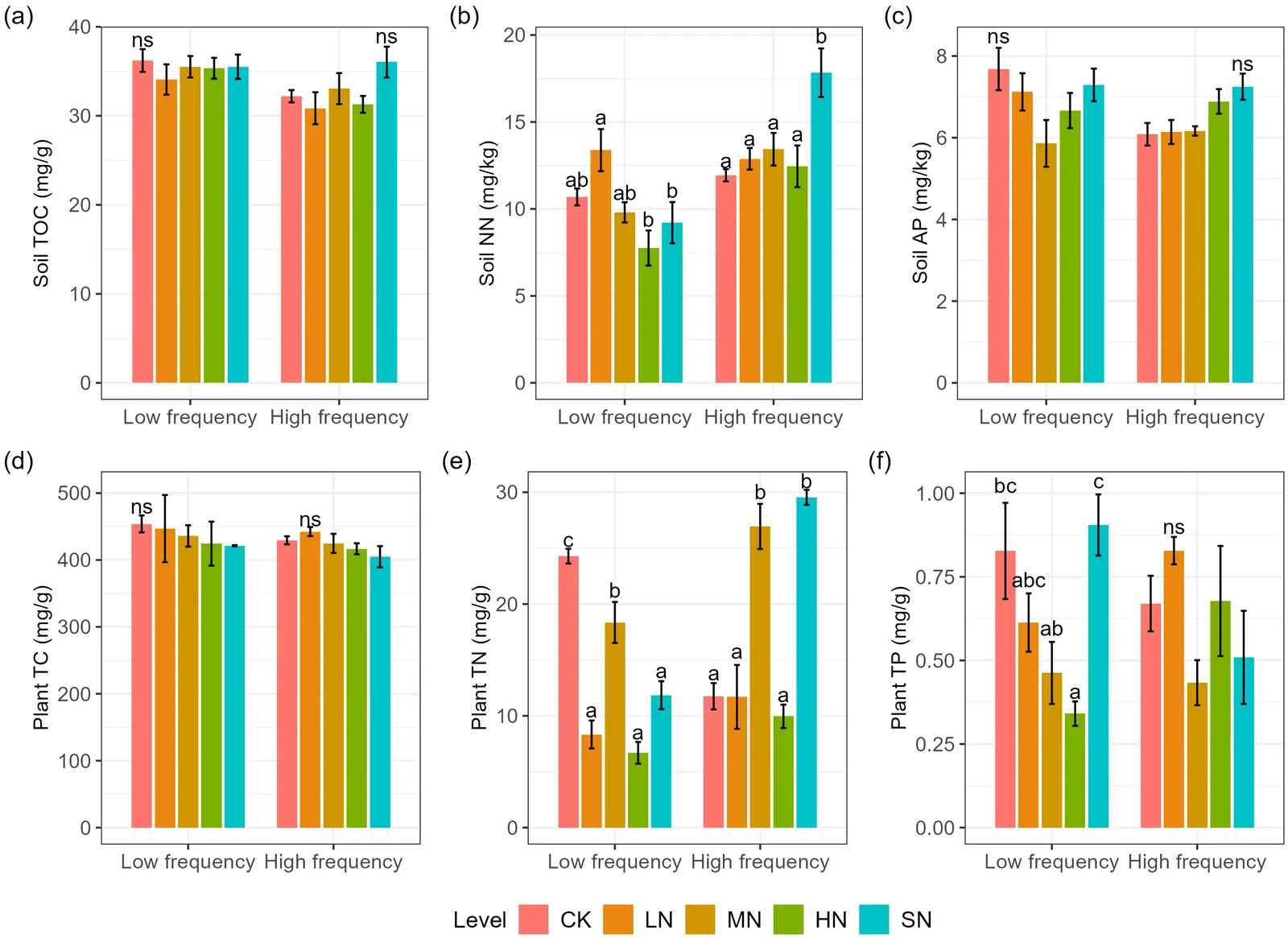
Effects of nitrogen addition level and frequency on key soil properties and plant nutrient concentrations: **(a)** soil total organic carbon (Soil TOC), **(b)** soil nitrate-N (Soil NN), **(c)** soil available phosphorus (Soil AP), **(d)** plant total carbon (Plant TC), **(e)** plant total nitrogen (Plant TN), and **(f)** plant total phosphorus (Plant TP). Bars represent means ± SE (n = 4). Different lowercase letters indicate significant differences among nitrogen addition levels within each frequency at p< 0.05 (one-way ANOVA, Tukey’s HSD). “ns” denotes no significant differences among levels within a given frequency. CK, control; LN, low nitrogen addition; MN, medium nitrogen addition; HN, high nitrogen addition; SN, severe nitrogen addition.

A randomized block design was used, with NH_4_NO_3_ (35% N) as the sole nitrogen source to simulate nitrogen deposition *in situ* (**Figure 2**). Five nitrogen addition levels were established: Control (CK; 0 g·m^-2^), Low N (LN; 5 g·m^-2^), Medium N (MN; 10 g·m^-2^), High N (HN; 15 g·m^-2^), and Severe N (SN; 20 g·m^-2^). These were combined with two addition frequencies: low-frequency and high-frequency application. Each treatment was replicated four times, for a total of 40 plots (3 m × 3 m each), separated by 0.5 m buffer strips. Low-frequency addition was applied once in late April 2021 (green-up stage). High-frequency addition was applied in late April, May, and June (green-up, jointing, and early flowering stages) to align with the active growth period of the dominant species, allowing sufficient time for nitrogen uptake and assimilation before the peak biomass sampling in late August.

### 2.3 Experimental methods

Plant sampling: in late August 2021, one 0.5 m × 0.5 m subplot was randomly positioned within each of the 40 plots. All aboveground plant material was harvested at ground level, sorted by species, and dried at 105 °C for 30 min, then at 65 °C for 48 h to constant weight. Dry biomass was weighed to 0.01 g, and samples were ground for determination of plant total organic carbon (plant TC), total nitrogen (plant TN), and total phosphorus (plant TP).

Soil sampling: After plant harvesting, surface litter was removed. Soil samples were collected using a soil auger (5 cm diameter) from three locations per plot and divided into two depths: surface layer (0–10 cm) and subsurface layer (10–20 cm). Soil samples from the same depth were pooled into one composite sample per plot, yielding 80 samples (40 plots × 2 depths). Each composite sample was split into two portions: one for analysis of soil water content (SWC), soil pH, soil salt content (SSC), soil total organic carbon (TOC), soil total nitrogen (soil TN), soil total phosphorus (soil TP), and soil available phosphorus (soil AP); the other was stored at 4 °C for later analysis of soil ammonium nitrogen (soil AN) and soil nitrate nitrogen (soil NN).

All analyses followed standard methods described in Soil Agrochemical Analysis (Bao, 2000) (**Table 1**).

### 2.4 Data handling

Data were compiled using Microsoft Excel 2010. Statistical analyses were performed with IBM SPSS Statistics 24.0 (IBM Corp., Armonk, NY, USA), and graphs were generated using R version 4.2.3 (R Core Team). Community ANPP was calculated as the sum of Poaceae, Rosaceae, and Fabaceae aboveground biomass for each plot. For soil properties, samples from the 0–10 cm and 10–20 cm layers were combined to obtain a single 0–20 cm composite sample per plot for all analyses. One-way analysis of variance (ANOVA) was applied to evaluate the individual effects of nitrogen addition level and the individual effects of addition frequency on soil physicochemical properties, plant aboveground productivity, and nutrient characteristics. Two-way ANOVA was employed to assess the main effects of nitrogen addition level and frequency, as well as their interaction.

For soil samples, the carbon-to-nitrogen (C:N), carbon-to-phosphorus (C:P), and nitrogen-to-phosphorus (N:P) ratios were calculated for each replicate (Equation 1):

(1) $$C:N_{soil,i}=\frac{Soil\;TOC_{i}}{Soil\;TN_{i}},\;C:P_{soil,i}=\frac{Soil\;TOC_{i}}{Soil\;TP_{i}},\;N:P_{soil,i}=\frac{Soil\;TN_{i}}{Soil\;TP_{i}}$$

where *Soil TOC_i_*, *Soil TN_i_*, and *Soil TP_i_* are the soil total organic carbon, total nitrogen, and total phosphorus concentrations of replicate *i* (n=4 per treatment).

For plant samples, the same stoichiometric ratios were calculated using plant TC, plant TN, and plant TP (Equation 2):

(2) $$C:N_{plant,i}=\frac{Plant\;TC_{i}}{Plant\;TN_{i}},\;C:P_{plant,i}=\frac{Plant\;TC_{i}}{Plant\;TP_{i}},\;N:P_{plant,i}=\frac{Plant\;TN_{i}}{Plant\;TP_{i}}$$

where *Plant TC_i_*, *Plant TN_i_*, and *Plant TP_i_* are the plant total carbon, total nitrogen, and total phosphorus concentrations of replicate *i* (n=4 per treatment).

Correlations between plant aboveground productivity, soil properties and nutrient traits were evaluated using Mantel tests and Pearson’s correlation analysis. A random forest model was constructed to identify key factors influencing aboveground productivity across functional groups; variables with high importance and significant correlations were retained as candidates. Based on the RFM and correlation results, separate SEMs were constructed for each plant functional group and each nitrogen addition frequency, yielding six independent models, to elucidate the pathways through which the selected factors exert their effects. This hierarchical framework ensures that the SEM is both statistically justified and ecologically meaningful.

## 3 Results

### 3.1 Effect of level and frequency of nitrogen addition on plant aboveground productivity and plant nutrient content

Both level and frequency, as well as their interaction, significantly affected plant nutrient contents and stoichiometric ratios across all groups. The responses of aboveground productivity to nitrogen addition varied among plant functional groups (**Table 2**). Below we detail the responses by community and functional group, with emphasis on level-dependent trends.

**Table 2:** Effects of nitrogen addition level and frequency on plant aboveground productivity and plant nutrient content.

Functional groups	Frequency	Level	Aboveground productivity (g/m^2^)	Plant TC (mg/g)	Plant TN (mg/g)	Plant TP (mg/g)	C: N	C:P	N:P
Poaceae	low-frequency	CK	80.14 ± 33.68a	465.99 ± 21.65a	21.91 ± 1.25c	0.56 ± 0.03ab	21.34 ± 1.93a	829.88 ± 53.48a	38.96 ± 1.35c
		LN	103.33 ± 25.34a	491.9 ± 118.7a	6.24 ± 2.52ab	0.48 ± 0.17ab	90.55 ± 45.89a	1088.04 ± 317.89a	12.9 ± 2.86a
		MN	92.81 ± 21.45a	465.05 ± 17.55a	11.2 ± 2.72ab	0.33 ± 0.02a	43.04 ± 8.53a	1411.73 ± 62.72a	34.14 ± 9.1bc
		HN	117.16 ± 31.89a	473.18 ± 68.19a	5.81 ± 4.25a	0.41 ± 0.16ab	193.61 ± 254.55a	1266.58 ± 486.61a	12.81 ± 6.44a
		SN	117.44 ± 18.27a	458.58 ± 4.39a	12.61 ± 3.36b	0.69 ± 0.24b	38.72 ± 12.1a	750.08 ± 346.89a	21.15 ± 11.91ab
	high-frequency	CK	82.29 ± 17.6a	416.8 ± 57.45a	7.82 ± 1.7a	0.81 ± 0.50a	55.13 ± 12.76a	644.62 ± 323.19a	11.64 ± 4.59a
		LN	75.2 ± 27.69a	485.08 ± 24.85a	10.60 ± 2.99a	0.44 ± 0.21a	48.68 ± 13.96a	1507.18 ± 1148.79a	39.68 ± 44.27a
		MN	116.05 ± 30.42ab	453.2 ± 27.39a	31.33 ± 7.80b	0.36 ± 0.23a	15.20 ± 3.96b	1646.69 ± 861.84a	101.97 ± 33.2a
		HN	109.47 ± 14.66ab	407.44 ± 34.63a	8.02 ± 1.97a	0.95 ± 0.76a	52.86 ± 11.44a	665.28 ± 478.65a	13.54 ± 12.05a
		SN	132.1 ± 14.53b	414.85 ± 37.61a	34.36 ± 2.06b	0.79 ± 0.47a	12.11 ± 1.32b	1011.27 ± 1150.65a	91.7 ± 114.49a
Rosaceae	low-frequency	CK	22.84 ± 15.59a	447.92 ± 8.51a	18.64 ± 5.19b	1.01 ± 0.23b	25.9 ± 9.06a	463.83 ± 123.9b	19.46 ± 8.35ab
		LN	27.26 ± 9.07a	423.67 ± 97.00a	7.17 ± 1.78a	1.07 ± 0.54b	60.04 ± 9.79a	529.79 ± 378.15b	8.38 ± 4.88a
		MN	37.34 ± 11.55a	454.63 ± 11.07a	20.47 ± 2.76b	0.65 ± 0.29ab	22.57 ± 3.53a	939.49 ± 703.65b	39.14 ± 22.2b
		HN	40.29 ± 13.67a	416.58 ± 62.04a	5.85 ± 1.95a	0.23 ± 0.07a	76.55 ± 26.77a	1921 ± 579.48a	26.13 ± 8.01ab
		SN	41.29 ± 16.61a	431.54 ± 20.18a	8.98 ± 5.44a	0.88 ± 0.14ab	67.24 ± 48.65a	495.93 ± 60.78b	9.87 ± 4.77a
	high-frequency	CK	24.59 ± 5.58a	421.78 ± 12.02a	12.86 ± 2.28a	0.96 ± 0.32a	33.8 ± 7.57a	475.09 ± 140.88a	14.15 ± 3.94a
		LN	27.77 ± 12.51a	433.35 ± 1.30a	13.05 ± 7.57a	0.93 ± 0.31a	44.74 ± 30.07a	525.78 ± 245.32a	18.81 ± 19.37a
		MN	40.01 ± 16.41a	417.18 ± 32.49a	23.11 ± 2.74b	0.63 ± 0.27a	18.3 ± 2.99a	752.2 ± 299.43a	41.94 ± 16.79ab
		HN	33.89 ± 11.59a	401.05 ± 26.67a	8.52 ± 2.27a	0.46 ± 0.27a	51.12 ± 20.54a	1101.31 ± 552.89a	21.48 ± 9.03a
		SN	34.98 ± 6.44a	391.56 ± 40.65a	24.12 ± 2.58b	0.42 ± 0.31a	16.32 ± 1.98a	1393.55 ± 920.99a	81.7 ± 47.4b
Fabaceae	low-frequency	CK	28.4 ± 19.96a	447.94 ± 85.11a	32.29 ± 2.57c	0.91 ± 0.86a	14.08 ± 3.70b	1598.59 ± 2091.5a	114.36 ± 157.92a
		LN	8.44 ± 7.02a	425.26 ± 96.66a	11.59 ± 6.02a	0.29 ± 0.09a	44.25 ± 19.72ab	1562.19 ± 399.6a	43.85 ± 27.25a
		MN	16.42 ± 16.73a	388.21 ± 73.79a	23.39 ± 7.05bc	0.41 ± 0.35a	16.98 ± 1.84b	1408.26 ± 828.03a	79.8 ± 40.28a
		HN	21.37 ± 7.86a	383.71 ± 73.67a	8.41 ± 3.29a	0.38 ± 0.18a	51.09 ± 21.45a	1098.12 ± 278.31a	25.18 ± 15.39a
		SN	22.44 ± 12.76a	373.88 ± 11.71a	13.95 ± 4.47ab	1.14 ± 0.45a	29.80 ± 12.87ab	373.73 ± 162.41a	14.87 ± 9.09a
	high-frequency	CK	16.91 ± 4.23a	449.78 ± 35.38a	14.58 ± 7.36ab	0.24 ± 0.16b	39.59 ± 26.45a	2665.86 ± 1941.71a	65.02 ± 11.65ab
		LN	18.58 ± 13.39a	409.03 ± 19.71a	11.43 ± 10.13a	1.11 ± 0.21a	63.22 ± 49.31a	378.2 ± 75.43a	10.35 ± 9.33a
		MN	16.24 ± 12.14a	404.08 ± 27.27a	26.39 ± 2.55bc	0.31 ± 0.22b	15.44 ± 2.08a	1796.23 ± 1038.45a	124.27 ± 84.42b
		HN	19.06 ± 7.18a	441.89 ± 32.00a	13.32 ± 3.83ab	0.62 ± 0.39ab	35.61 ± 11.47a	957.94 ± 570.72a	33.04 ± 28.81ab
		SN	20.69 ± 5.72a	408.32 ± 36.33a	30.16 ± 5.34c	0.32 ± 0.15b	13.98 ± 3.36a	1731.34 ± 1335.11a	118.7 ± 66.79ab

Different lowercase letters within the same functional group, same frequency (Low or High), and same variable column indicate significant differences among nitrogen addition levels (CK, LN, MN, HN, SN) at p< 0.05. Levels sharing the same letter are not significantly different.

ANPP responded differently to increasing nitrogen addition levels under both low- and high-frequency treatments (**Figure 1**). Community-level ANPP increased with N addition level under both frequencies (**Table 2**), with a significant main effect of level (F = 10.930, P< 0.001; **Table 3**), while frequency had no significant effect (F = 0.073, P = 0.789; **Table 3**). The level × frequency interaction was also not significant (F = 1.448, P = 0.243; **Table 3**). Poaceae aboveground productivity increased with nitrogen addition level under both frequencies, with a significant effect under high-frequency addition (p< 0.05). Two-way ANOVA confirmed that nitrogen addition level had a significant main effect on Poaceae ANPP (p = 0.009), whereas frequency did not (**Table 3**). Low-frequency addition significantly influenced plant TN and TP, and high-frequency addition affected plant TC, TN, and TP. Stoichiometric ratios (C:N, C:P, N:P) responded to both frequencies. In contrast, Rosaceae and Fabaceae aboveground productivity showed no significant response to either level or frequency (**Table 2**). However, their nutrient contents (plant TN, TP) and stoichiometric ratios (C:N, C:P, N:P) were significantly altered by level, frequency, and their interaction (**Table 3**). The N:P ratios of Rosaceae and Fabaceae increased markedly under high-level treatments and high-frequency (e.g., Fabaceae N:P reached 124.27 at MN and 118.70 at SN), indicating progressive phosphorus limitation. Within each nitrogen addition level, the effect of addition frequency on aboveground productivity was relatively limited. For instance, in Poaceae, significant differences between low- and high-frequency treatments were only observed at specific nitrogen levels, whereas in Rosaceae and Fabaceae, frequency effects on productivity were generally non-significant. These results indicate that nitrogen addition level, rather than frequency, plays a dominant role in regulating aboveground productivity, particularly in Poaceae.

**Table 3:** Effects of nitrogen addition level and frequency on plant aboveground productivity and plant nutrient content.

Plant		Aboveground productivity		Plant TC		Plant TN		Plant TP		C: N		C:P		N:P	
Functional groups	N addition	F	P	F	P	F	P	F	P	F	P	F	P	F	P
Poaceae	level	4.139	**0.009**	1.385	0.263	35.091	**<0.000**	1.858	0.144	11.060	**<0.000**	2.635	0.054	20.257	**<0.000**
	frequency	0.012	0.914	4.690	**0.038**	37.658	**<0.000**	2.422	0.130	6.181	**0.019**	1.150	0.292	7.512	**0.010**
	level*frequency	1.335	0.280	0.476	0.753	34.486	**<0.000**	0.840	0.511	10.540	**<0.000**	2.146	0.100	9.856	**<0.000**
Rosaceae	level	2.465	0.066	0.764	0.557	17.197	**<0.000**	6.766	**0.001**	6.000	**0.001**	3.570	**0.017**	11.968	**<0.000**
	frequency	0.156	0.696	2.749	0.108	11.061	**0.002**	0.878	0.356	13.145	**0.001**	0.313	0.58	12.502	**0.001**
	level*frequency	0.255	0.905	0.466	0.760	7.425	**<0.000**	1.403	0.257	3.552	**0.017**	3.078	**0.031**	12.732	**<0.000**
Fabaceae	level	0.848	0.506	1.279	0.300	11.221	**<0.000**	1.279	0.300	6.730	**0.001**	2.617	0.055	7.274	**<0.000**
	frequency	0.091	0.766	1.096	0.304	0.474	0.496	0.789	0.381	0.258	0.615	0.733	0.399	3.491	0.071
	level*frequency	0.858	0.500	0.513	0.726	9.127	**<0.000**	6.524	**0.001**	1.932	0.131	2.152	**0.099**	5.097	**0.003**

Bold values indicate statistically significant effects (p < 0.05).

The lack of a significant level or frequency effect on Rosaceae and Fabaceae ANPP suggests that these functional groups are less responsive to nitrogen enrichment under the conditions of this short-term experiment.

### 3.2 Effect of level and frequency of nitrogen addition on soil physicochemical properties

Two-way ANOVA revealed significant interactions between nitrogen addition level and frequency for SSC, soil TOC, soil NN, and soil AP, as well as a significant main effect of level on AP in the 0–10 cm layer (p = 0.049; **Supplementary Table 1**). No significant interaction was found for soil pH (p > 0.05; **Supplementary Table 1**), indicating that addition frequency did not mitigate the acidifying effect of nitrogen addition. In contrast, nitrogen addition frequency alone had no significant main effect on most soil variables (**Supplementary Table 1**). These results indicate that nitrogen addition level, together with its interaction with frequency, is the primary driver of changes in soil physicochemical properties.

One-way ANOVA (**Supplementary Table 1**) further showed that when analyzed separately, nitrogen addition level significantly affected soil NN only in the 10–20 cm layer (p = 0.008). Nitrogen addition frequency significantly affected soil NN in both the 0–10 cm and 10–20 cm layers (p< 0.001 and p = 0.032, respectively) and soil TOC in the 10–20 cm layer (p = 0.025). Neither level nor frequency had significant effects on other soil variables in this isolated analysis. These results underscore the importance of considering both factors and their interaction, as captured by the two-way ANOVA.

For completeness, we also present the effects of nitrogen addition on soil physicochemical properties (**Table 4**). Under low-frequency addition, significant effects were observed on soil NN, soil AP, and soil AN in the 0–10 cm layer, and on soil SSC, soil TOC, soil NN, and soil AP in the 10–20 cm layer. Under high-frequency addition, significant effects were found on soil NN, soil SSC, soil TOC, and soil TP in the 0–10 cm layer, and on soil pH, soil NN, and soil N:P in the 10–20 cm layer.

**Table 4:** Effects of nitrogen addition on soil physicochemical properties.

Soil layers	Frequency	Level	SWC(%)	pH	SSC(mg/g)	Soil TOC(mg/g)	Soil TN(mg/g)	Soil AN(mg/kg)	Soil NN(mg/kg)	Soil TP(mg/g)	Soil AP(mg/kg)	C:N	C:P	N:P
0–10 cm	low-frequency	**CK**	14.75 ± 2.06a	7.98 ± 0.14a	0.69 ± 0.07a	40.21 ± 5.27a	3.93 ± 2.49a	8.90 ± 1.04a	11.15 ± 1.42ab	0.40 ± 0.18a	9.58 ± 1.47a	20.84 ± 25.12a	128.59 ± 86.25a	15.31 ± 18.69a
		LN	13.50 ± 2.38a	7.94 ± 0.16a	0.79 ± 0.06a	41.58 ± 6.08a	3.16 ± 1.24a	8.29 ± 0.65ab	15.01 ± 3.36a	0.53 ± 0.20a	9.28 ± 1.40a	15.32 ± 8.10a	83.26 ± 19.24a	7.02 ± 4.19a
		MN	14.25 ± 0.50a	8.02 ± 0.12a	0.73 ± 0.10a	39.35 ± 3.25a	2.09 ± 2.51a	7.67 ± 0.42b	9.54 ± 1.83b	0.46 ± 0.08a	7.04 ± 1.70b	71.77 ± 79.78a	87.77 ± 20.36a	4.37 ± 5.45a
		HN	13.75 ± 1.26a	7.98 ± 0.06a	0.71 ± 0.11a	35.44 ± 6.13a	1.53 ± 1.82a	7.69 ± 0.95b	8.39 ± 4.00b	0.42 ± 0.16a	8.24 ± 1.27ab	46.28 ± 27.95a	99.31 ± 48.48a	3.49 ± 3.03a
		SN	14.25 ± 4.03a	7.95 ± 0.07a	0.67 ± 0.06a	39.45 ± 4.86a	1.22 ± 0.87a	8.06 ± 0.69ab	10.37 ± 4.06ab	0.47 ± 0.12a	8.92 ± 0.69ab	54.83 ± 45.32a	89.26 ± 27.74a	2.92 ± 2.34a
	high-frequency	CK	14.50 ± 1.29a	7.82 ± 0.34a	0.76 ± 0.02b	32.21 ± 0.48a	2.74 ± 2.20a	6.72 ± 0.92a	10.23 ± 0.52ab	0.39 ± 0.23ab	5.78 ± 0.92a	61.77 ± 101.80a	209.87 ± 288.33a	13.06 ± 15.13a
		LN	14.00 ± 2.16a	8.02 ± 0.05a	0.68 ± 0.04b	26.58 ± 1.17b	2.15 ± 1.21a	7.90 ± 4.25a	11.76 ± 3.25a	0.26 ± 0.27b	4.96 ± 0.97a	22.21 ± 24.45a	492.81 ± 609.91a	22.35 ± 22.76a
		MN	14.25 ± 1.26a	8.01 ± 0.13a	0.74 ± 0.06b	31.67 ± 2.11a	3.12 ± 1.74a	6.41 ± 0.61a	10.06 ± 1.67ab	0.44 ± 0.23ab	4.68 ± 0.80a	13.08 ± 7.00a	93.83 ± 58.62a	11.11 ± 11.35a
		HN	13.50 ± 1.29a	7.99 ± 0.43a	0.75 ± 0.05b	35.24 ± 5.38a	1.58 ± 1.28a	5.64 ± 1.25a	7.12 ± 0.78c	0.61 ± 0.09a	5.09 ± 0.82a	44.67 ± 42.24a	59.48 ± 16.64a	2.41 ± 1.68a
		SN	13.25 ± 1.26a	7.94 ± 0.18a	0.86 ± 0.08a	31.57 ± 0.76a	1.54 ± 0.68a	5.97 ± 1.51a	8.05 ± 0.76bc	0.45 ± 0.18ab	5.66 ± 1.07a	26.27 ± 18.10a	81.48 ± 36.23a	3.93 ± 2.76a
10–20 cm	low-frequency	CK	14.74 ± 1.29a	8.11 ± 0.05a	0.66 ± 0.06b	35.84 ± 2.41b	1.88 ± 2.99a	7.62 ± 0.91a	12.43 ± 0.45b	0.50 ± 0.30a	7.17 ± 1.71b	76.68 ± 53.48a	96.62 ± 56.04a	3.34 ± 4.52a
		LN	13.87 ± 2.14a	8.05 ± 0.14a	0.81 ± 0.13a	34.46 ± 4.95b	2.78 ± 3.42a	8.99 ± 1.58a	13.77 ± 3.21b	0.51 ± 0.21a	7.61 ± 0.91b	28.71 ± 19.95a	73.53 ± 21.31a	7.52 ± 11.38a
		MN	14.16 ± 1.06a	8.10 ± 0.12a	0.74 ± 0.08ab	35.82 ± 1.74b	2.09 ± 0.51a	7.81 ± 0.86a	17.61 ± 3.26ab	0.47 ± 0.25a	7.31 ± 0.91b	17.86 ± 4.24a	102.08 ± 66.45a	5.88 ± 3.74a
		HN	13.58 ± 1.34a	8.03 ± 0.18a	0.68 ± 0.05ab	34.99 ± 2.60b	1.46 ± 0.85a	9.22 ± 1.67a	16.16 ± 5.48ab	0.32 ± 0.13a	8.61 ± 1.11ab	33.49 ± 23.45a	131.86 ± 77.69a	6.08 ± 5.8a
		SN	13.04 ± 0.99a	8.07 ± 0.21a	0.70 ± 0.10ab	42.48 ± 4.15a	3.00 ± 3.26a	7.78 ± 0.50a	21.66 ± 5.75a	0.45 ± 0.22a	9.37 ± 0.35a	68.43 ± 104.87a	116.19 ± 56.93a	8.44 ± 12.07a
	high-frequency	CK	16.25 ± 2.63a	8.18 ± 0.04a	0.72 ± 0.06a	28.57 ± 2.00a	2.14 ± 2.88a	6.22 ± 0.36a	11.45 ± 1.36bc	0.51 ± 0.19a	5.01 ± 0.80a	108.02 ± 159.34a	62.03 ± 22.23a	3.72 ± 3.88b
		LN	15.50 ± 4.51a	7.84 ± 0.27b	0.78 ± 0.10a	27.25 ± 3.60a	1.79 ± 1.60a	5.76 ± 0.53a	12.00 ± 1.18ab	0.40 ± 0.15a	4.67 ± 0.50a	32.98 ± 29.55a	78.06 ± 34.35a	6.99 ± 9.09ab
		MN	14.75 ± 0.96a	8.10 ± 0.03a	0.74 ± 0.7a	30.30 ± 5.29a	1.66 ± 1.73a	5.78 ± 1.62a	9.25 ± 1.01cd	0.53 ± 0.31a	5.03 ± 0.53a	33.45 ± 21.44a	77.47 ± 47.96a	5.41 ± 6.76ab
		HN	13.75 ± 0.96a	8.16 ± 0.04a	0.87 ± 0.28a	27.58 ± 1.20a	2.61 ± 1.98a	5.58 ± 1.01a	8.75 ± 0.89d	0.48 ± 0.32a	5.17 ± 0.41a	16.06 ± 10.53a	102.63 ± 102.4a	6.72 ± 4.34ab
		SN	14.75 ± 1.50a	8.12 ± 0.04a	0.79 ± 0.02a	29.79 ± 5.73a	3.60 ± 1.82a	7.58 ± 3.11a	14.00 ± 2.51a	0.20 ± 0.13a	5.13 ± 1.18a	10.03 ± 4.97a	331.65 ± 403.82a	41.76 ± 54.72a

Different lowercase letters within the same soil layer, same frequency (Low or High), and same variable column indicate significant differences among nitrogen addition levels (CK, LN, MN, HN, SN) at p< 0.05. Levels sharing the same letter are not significantly different.

In addition to these tabulated results, the responses of key soil properties (soil TOC, NN, and AP) and plant nutrient concentrations (plant TC, TN, and TP) to nitrogen addition level and frequency are illustrated in **Figure 2**. Soil TOC and NN showed significant level × frequency interactions, while AP exhibited a significant level main effect. These coordinated changes in soil nutrient availability and plant nutrient status provide mechanistic insights into how nitrogen addition regulates aboveground productivity, which is further explored in the following section.

### 3.3 Correlation analysis between plant aboveground productivity and soil physicochemical properties

Nitrogen addition level fundamentally altered plant aboveground productivity and soil properties, and the strength of correlations between them. Pearson’s correlation analysis was applied to assess relationships among soil physicochemical properties. A soil property matrix was constructed based on physical and chemical attributes, while a plant community matrix was derived from the aboveground productivity of different functional groups. Mantel tests were used to evaluate the correlation between plant aboveground productivity and soil properties.

Under low-frequency addition, Pearson correlation revealed that soil TOC was negatively correlated with soil pH (p< 0.05). Soil AN was positively correlated with SSC and negatively correlated with soil TP. Soil NN was positively correlated with soil TOC, significantly positively correlated with SSC (p< 0.01), and highly significantly positively correlated with soil AN (p< 0.001). Soil AP was positively correlated with soil NN, exhibited a highly significant positive correlation with soil TOC and soil AN, and was negatively correlated with soil pH. Soil C:N was significantly negatively correlated with soil TN, while soil C:P was highly significantly negatively correlated with soil TP. Soil N:P was positively correlated with soil NN, significantly positively correlated with soil TN and soil AN, and negatively correlated with soil TOC and C:N.

**Figure 3:**
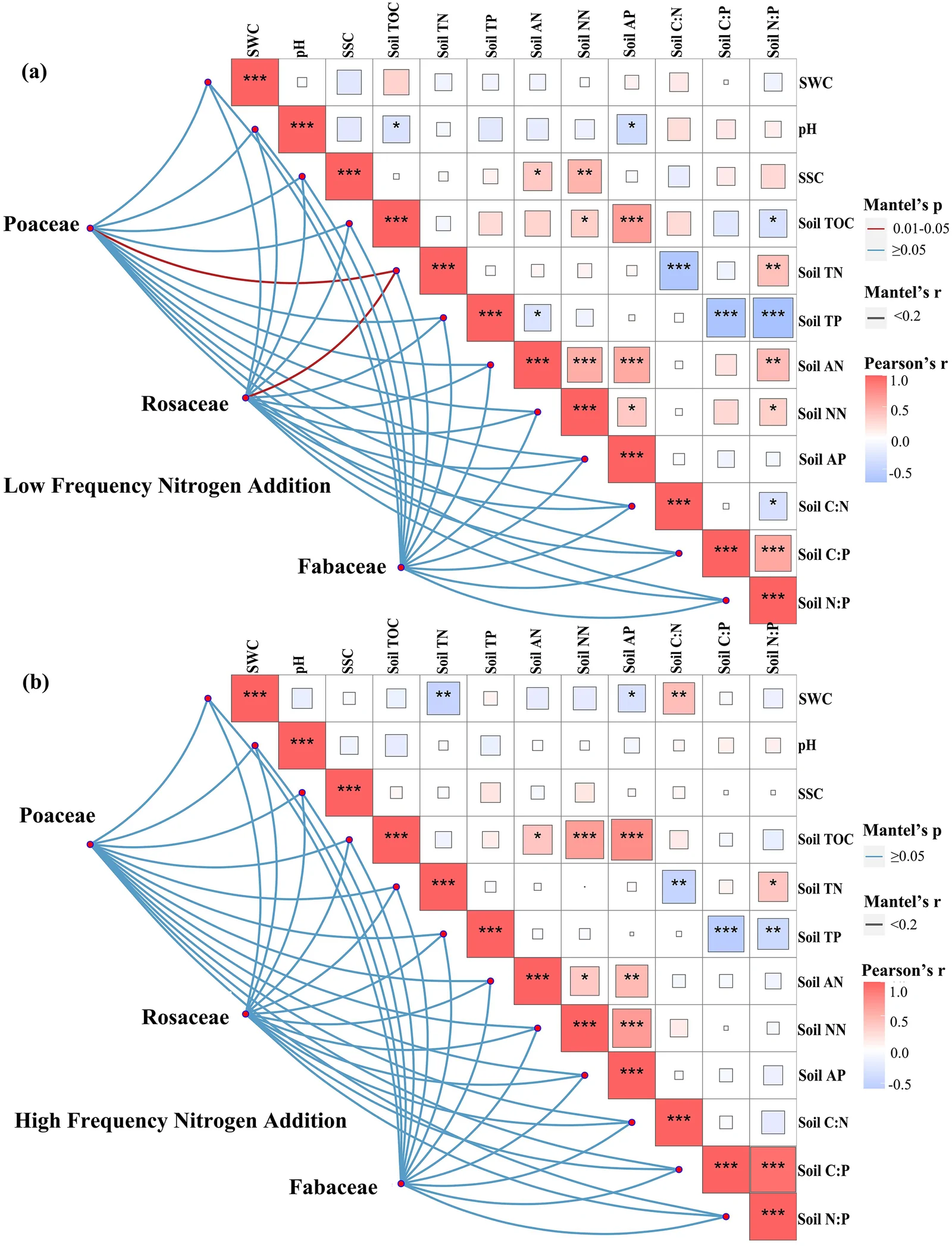
Correlation analysis between soil physicochemical properties and aboveground productivity under **(a)** low-frequency nitrogen addition and **(b)** high-frequency nitrogen addition. Half-matrix color gradient represents Pearson’s correlation coefficient. ***, **, * indicate significance at p< 0.001, 0.01, and 0.05, respectively. Mantel test results are shown as lines connecting soil properties to functional groups: line width corresponds to Mantel’s r, line color to significance level. Correlation coefficients for the three functional groups are shown in the table.

Mantel test results indicated that under low-frequency addition, the aboveground productivity of Poaceae and Rosaceae was correlated with soil TN (Mantel’s r< 0.2, p< 0.05), whereas Fabaceae productivity showed no correlation with soil properties (Mantel’s r< 0.2, p > 0.05; see **Figure 3**).

Under high-frequency addition, soil TN was significantly negatively correlated with SWC (p< 0.01). Soil AN was positively correlated with soil TOC; soil NN was positively correlated with soil AN (p< 0.05) and highly significantly positively correlated with soil TOC (p< 0.001). Soil AP showed significant positive correlation with soil AN, highly significant positive correlation with soil TOC and soil NN, and a negative correlation with SWC. Soil C:N was significantly positively correlated with SWC and negatively correlated with soil TN. Soil C:P was significantly negatively correlated with soil TP. Soil N:P was positively correlated with soil TN, significantly positively correlated with soil C:P, and significantly negatively correlated with soil TP. Mantel tests under high-frequency addition revealed no significant correlation between the aboveground productivity of any plant functional group and soil physicochemical properties (Mantel’s r< 0.2, p > 0.05).

These results indicate that the coupling between soil properties and plant productivity is strongly influenced by nitrogen addition level, with frequency modulating the strength of these relationships. The significant correlations observed under low-frequency addition disappeared under high-frequency addition, suggesting that more frequent nitrogen inputs decouple soil–plant nutrient linkages.

### 3.4 Correlation analysis between soil physicochemical properties and plant nutrient content

**Figure 4:**
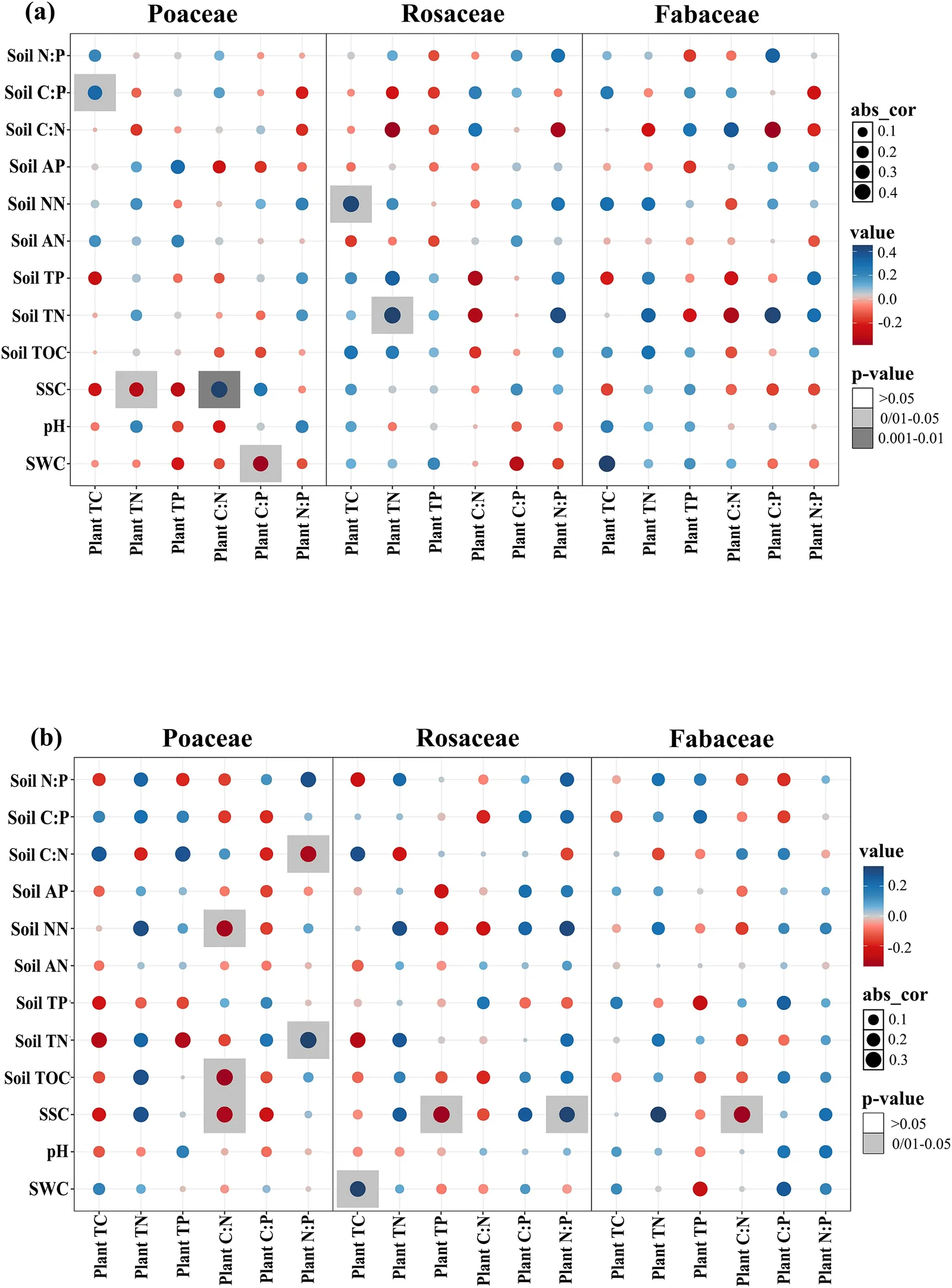
Correlation analysis between soil physicochemical properties and plant nutrient contents of **(a)** low-frequency nitrogen addition and **(b)** high-frequency nitrogen addition. Horizontal axis: plant nutrient content; vertical axis: soil properties. Color gradient represents Pearson’s correlation coefficient.

Correlations between plant nutrient content and soil environmental factors can indicate plant sensitivity to external conditions. Under low-frequency addition, significant correlations were observed in Poaceae and Rosaceae, but not in Fabaceae (**Figure 4**). Pearson correlation analysis revealed that in Poaceae, plant TC was positively correlated with soil C:P (p< 0.05), while plant TN was negatively correlated with SSC. Plant C:N showed a significant positive correlation with SSC (p< 0.01), and plant C:P was negatively correlated with SWC. In Rosaceae, plant TC was positively correlated with soil NN, and plant TN was positively correlated with soil TN. Overall, nutrient cycling in Poaceae was jointly influenced by SWC, SSC, and soil C:P, whereas in Rosaceae it was primarily associated with soil TN and soil NN.

Under high-frequency addition, nutrient contents of all three plant functional groups correlated with soil environmental factors. In Poaceae, plant C:N was negatively correlated with SSC, soil TOC, and soil NN (p< 0.05); plant N:P was positively correlated with soil TN and negatively correlated with soil C:N; plant TC was positively correlated with SSC and negatively correlated with soil TOC. In Rosaceae, plant TC was positively correlated with SWC; plant TP was negatively correlated with SSC; plant N:P was positively correlated with SSC. In Fabaceae, plant C:N was negatively correlated with SSC. Thus, nutrient turnover in Poaceae was influenced by SSC, TC, TN, NN, and C:N; in Rosaceae by SWC and SSC; and in Fabaceae primarily by SSC.

### 3.5 Analysis of drivers of aboveground productivity changes in plants of different functional groups

**Figure 5:**
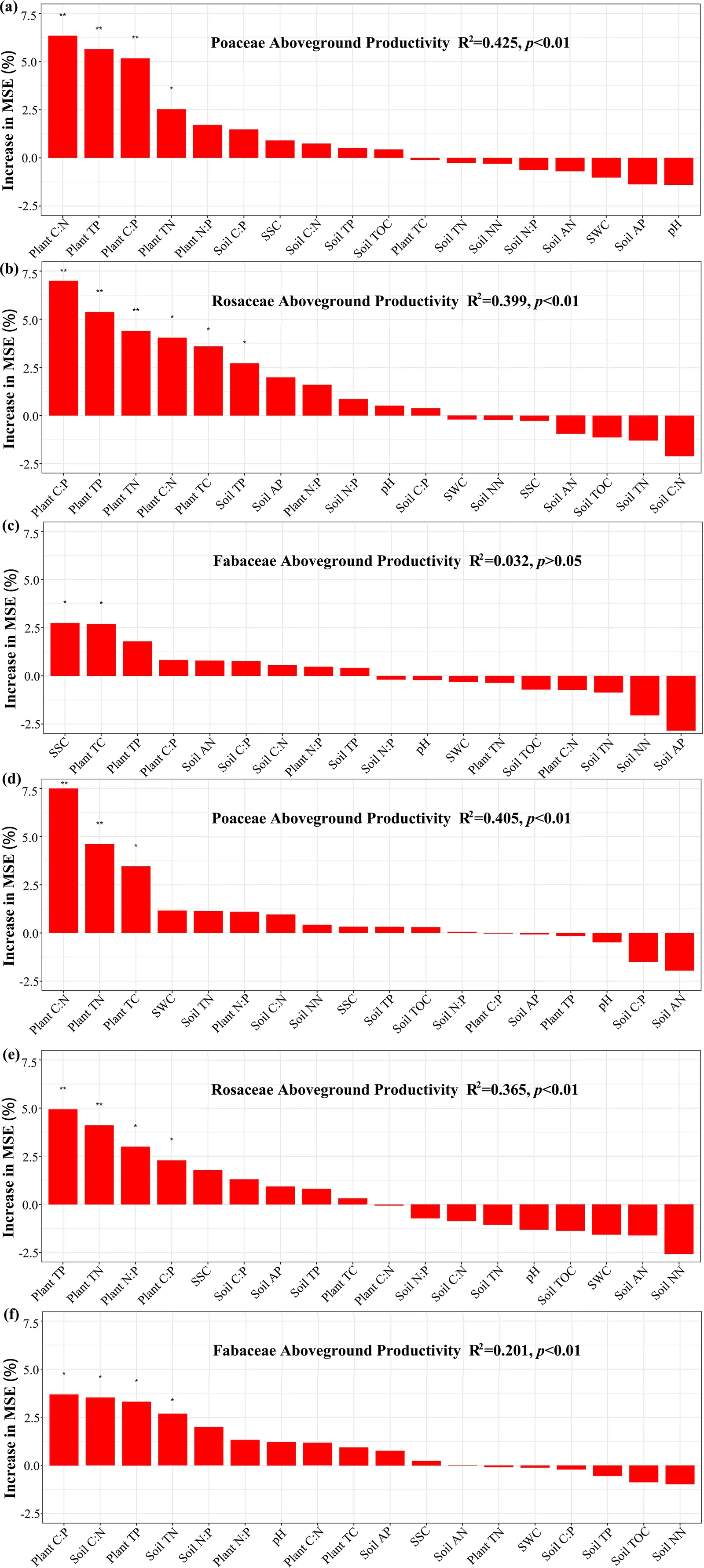
Drivers of **(a)** Poaceae, **(b)** Rosaceae, and **(c)** Fabaceae aboveground productivity changes under low-frequency nitrogen addition; and **(d)** Poaceae, **(e)** Rosaceae, and **(f)** Fabaceae aboveground productivity changes under high-frequency nitrogen addition. Variable importance measured as %IncMSE (percentage increase in mean squared error); higher values indicate greater importance. ***, **, * indicate significance at p< 0.001, 0.01, and 0.05, respectively.

Random forest model (RFM) was used to identify key predictors of aboveground productivity. The results revealed that significant factors varied between addition modes (**Figure 5**). Under low-frequency addition, RFM demonstrated strong explanatory power for Poaceae (R² = 0.425, p< 0.01) and Rosaceae (R² = 0.399, p< 0.05), but weak explanatory power for Fabaceae (R² = 0.032, p > 0.05) (**Figure 5**). For Poaceae, plant TP, plant C:N, and plant C:P had highly significant effects (p< 0.01), while plant TN had significant effects (p< 0.05). For Rosaceae, plant TN, plant TP, and plant C:P exhibited highly significant effects (p< 0.01), whereas plant TC, plant C:N, and soil TP showed significant effects (p< 0.05). For Fabaceae, plant TC and SSC were identified as significant factors (p< 0.05).

Under high-frequency addition, RFM demonstrated strong explanatory power for Poaceae (R² = 0.405, p< 0.01), Rosaceae (R² = 0.365, p< 0.05), and Fabaceae (R² = 0.201, p< 0.01). For Poaceae, plant TN and plant C:N had highly significant effects (p< 0.01), while plant TC had significant effects (p< 0.05). For Rosaceae, plant TN and plant TP had highly significant effects (p< 0.01), whereas plant C:P and plant N:P demonstrated significant effects (p< 0.05). For Fabaceae, plant TP, plant C:P, soil TN, and soil C:N were significant factors.

### 3.6 Analysis of pathways affecting changes in aboveground productivity of plants in different functional groups

**Figure 6:**
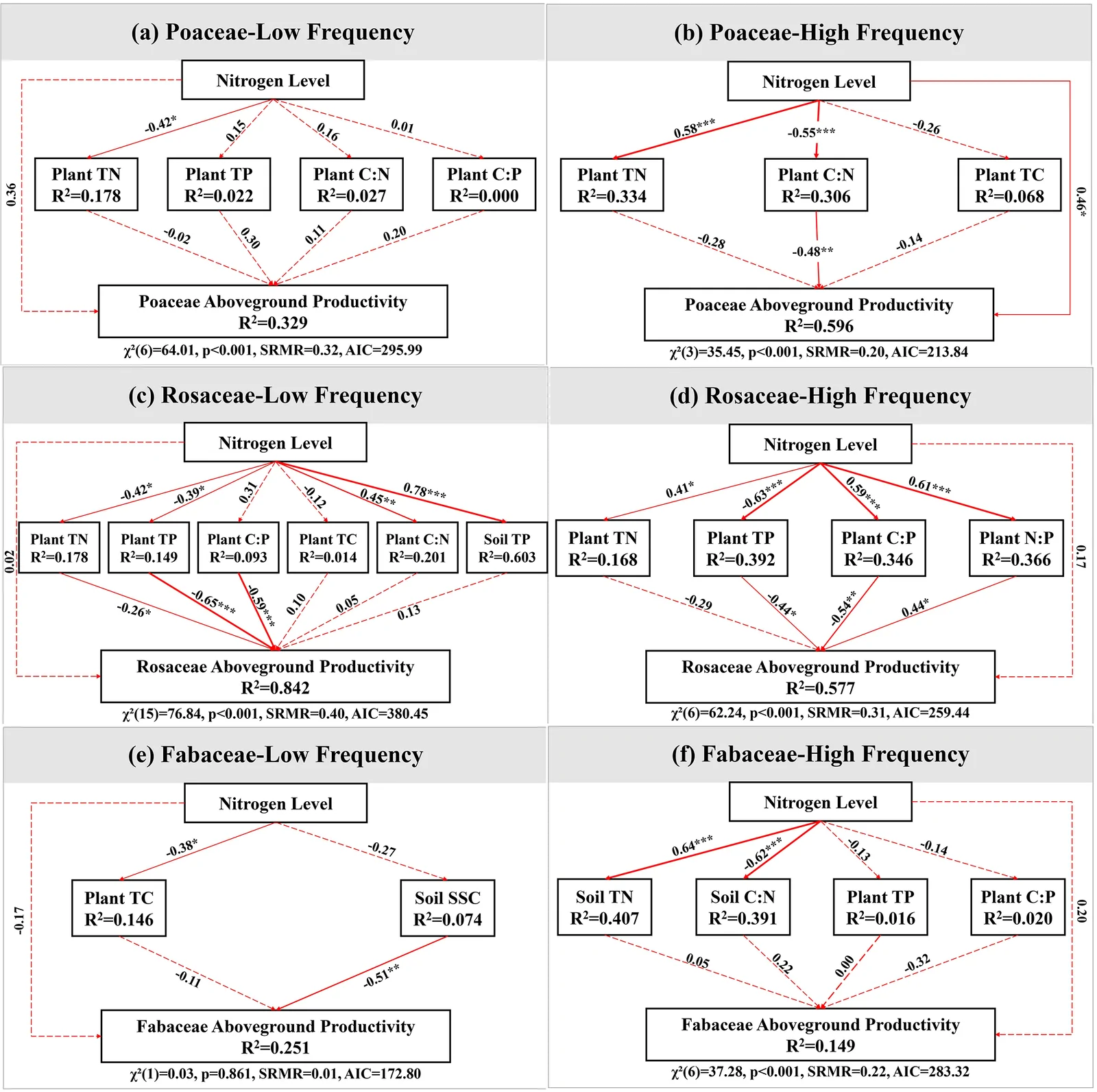
Pathways by which nitrogen addition affects aboveground productivity of Poaceae under **(a)** low frequency, **(b)** high frequency, Rosaceae under **(c)** low frequency, **(d)** high frequency, and Fabaceae under **(e)** low frequency, **(f)** high frequency. SEMs were constructed separately for each functional group and each nitrogen addition frequency based on RFM-selected predictors. Variables with significant paths (p< 0.05) are shown for clarity. Solid and dashed arrows indicate direct and indirect relationships, respectively; arrow width is proportional to path coefficient significance. ***, **, * indicate significance at p< 0.001, 0.01, and 0.05, respectively.

Based on RFM results, SEM was employed to analyze pathways through which nitrogen addition influences aboveground productivity in different functional groups. SEM results demonstrated both direct and indirect effects, with pathways varying substantially among functional groups and nitrogen addition frequency (**Figure 6**).

Nitrogen addition level directly affected the aboveground productivity in Poaceae under high frequency, but not in other groups. For Poaceae, under low-frequency addition, the direct path from nitrogen level to ANPP was not significant (p = 0.060). Addition level directly affected plant TN. The SEM explained 17.8%, 2.2%, 2.7%, 0%, and 32.9% of the variance in plant TN, TP, C:N, C:P, and ANPP, respectively. Under high-frequency addition, a significant direct positive effect of nitrogen level on ANPP emerged. Nitrogen level decreased plant C:N, which in turn negatively affected ANPP, constituting a significant indirect pathway. The SEM explained 33.4%, 30.6%, 6.8%, and 59.6% of the variance in plant TN, C:N, TC, and ANPP, respectively.

For Rosaceae, nitrogen level did not directly affect ANPP under either frequency (both p > 0.05). Under low-frequency addition, the SEM explained 17.8%, 14.9%, 9.3%, 1.3%, 20.1%, 60.3%, and 84.2% of the variance in plant TN, TP, C:P, TC, C:N, soil TP, and ANPP, respectively. Nitrogen addition level significantly affected plant TN, TP, C:N and soil TP. Plant TN, TP and C:P exerted strong negative effects on ANPP. Under high-frequency addition, the SEM explained 16.8%, 39.2%, 34.6%, 36.6%, and 57.7% of the variance in plant TN, TP, C:P, N:P, and ANPP, respectively. Nitrogen addition level directly influenced plant TN, TP, C:P, and N:P. Plant TP, C:P, and N:P all significantly affected ANPP.

For Fabaceae, no significant direct effect of nitrogen level on ANPP was detected under either frequency. Under low-frequency addition, the SEM explained 14.6%, 7.4%, and 25.1% of the variance in plant TC, SSC, and ANPP, respectively. Addition level directly affected plant TC, while SSC negatively affected ANPP. Under high-frequency addition, the SEM explained only 14.9% of ANPP variance, with addition level significantly affecting soil TN and soil C:N but neither soil property significantly predicted ANPP.

These findings demonstrate that the direct effect of nitrogen addition level on ANPP was significant only for Poaceae under high-frequency addition, with additional indirect mediation via plant C:N. For Rosaceae, nitrogen addition operated indirectly through phosphorus-related traits (plant TP, C:P and N:P) and plant TN, while for Fabaceae, no significant pathway from nitrogen level to ANPP was detected, indicating that Fabaceae ANPP is largely insensitive to nitrogen enrichment.

## 4 Discussion

### 4.1 Nitrogen addition regulates ANPP through alterations in soil properties and plant nutrients

Nitrogen is a key limiting resource for plant growth in most terrestrial ecosystems (LeBauer and Treseder, 2008), and its addition generally alleviates this limitation, promoting ANPP (Bai et al., 2010). In this study, ANPP responded positively to increasing nitrogen addition levels under both low- and high-frequency treatments, indicating that the total amount of nitrogen added is a primary driver of productivity in this alpine grassland.

The increase in ANPP was accompanied by significant changes in soil nutrient availability. Soil NN increased significantly under both frequencies, and AP increased significantly under low-frequency addition (see **Table 4**). Two-way ANOVA further revealed a significant main effect of nitrogen addition level on AP in the 0–10 cm layer (p = 0.049; see **Supplementary Table 5**), and significant level × frequency interactions for soil NN. These results suggest that nitrogen addition, particularly through increased nitrogen level, enhanced soil nutrient supply, thereby fueling aboveground biomass accumulation. The depth-dependent responses of TOC, NN, and AP (see **Table 4**) further indicate that surface soil nutrient enrichment is a key driver of ANPP.

Although nitrogen addition level played the dominant role, addition frequency also modulated soil properties in ways that indirectly affected ANPP. Previous studies have shown that nitrogen addition frequency can significantly alter soil chemical properties, including pH, base cations, and micronutrient availability (Wang et al., 2018; Li et al., 2019). In this study, one-way ANOVA (see **Table 2**) showed that when analyzed separately, frequency significantly affected NN in both soil layers and TOC in the 10–20 cm layer, whereas level alone affected only NN in the 10–20 cm layer. Moreover, Mantel tests revealed that under low-frequency addition, soil TN was correlated with the aboveground productivity of Poaceae and Rosaceae (Mantel’s r< 0.2, p< 0.05), whereas under high-frequency addition, no soil property was directly correlated with plant productivity (see **Figure 3**). This suggests that the coupling between soil properties and plant productivity is stronger under infrequent N pulses than under frequent pulses. The interaction between water and nitrogen availability is critical in semi-arid grasslands, where soil moisture strongly mediates nutrient transport and microbial activity (Harpole et al., 2007). However, the lack of a significant frequency main effect on ANPP for any of the three functional groups (**Table 3**) suggests that frequency did not independently alter the productivity of Poaceae, Rosaceae, or Fabaceae. Instead, frequency appears to act through finer soil–plant interactions, while nitrogen addition level remains the primary determinant of ANPP at the functional group level. Indeed, a recent study in temperate grasslands demonstrated that aboveground productivity depends more on the rate than on the frequency of nitrogen addition (Zhang et al., 2015).

Soil pH did not change significantly following nitrogen addition (**Supplementary Table 5**; **Table 4**). All pH values remained above 7.5, indicating that soil remained alkaline throughout the experiment. In contrast, SWC did not change significantly with nitrogen addition (**Table 4**), indicating that water limitation was not exacerbated under our experimental conditions and therefore did not constrain the positive ANPP response. Taken together, our findings demonstrate that nitrogen addition level is the primary driver of ANPP in this alpine grassland, primarily through increasing soil NN and AP availability. Addition frequency plays a secondary, indirect role by modulating soil–plant nutrient coupling, but does not independently affect total productivity.

### 4.2 Nitrogen addition exacerbates plant P limitation and reduces ANPP in non-grass species

Aboveground productivity varied markedly among functional groups. Poaceae showed significantly higher ANPP than Rosaceae and Fabaceae, as well as a greater responsiveness to nitrogen addition (see **Table 2**). Two-way ANOVA confirmed that nitrogen addition level had a significant main effect on Poaceae ANPP (p = 0.009), whereas frequency did not (see **Table 3**). This pattern aligns with the well-documented competitive advantages of graminoids under nitrogen enrichment: their rapid growth rates, tall stature, and efficient nutrient acquisition strategies allow them to pre-empt light and soil resources more effectively than forbs and legumes (Bai et al., 2010; Stevens et al., 2006). Exogenous nutrient inputs shift plant competition from nutrient limitation to light limitation (Li et al., 2011); as a result, the morphological traits of Poaceae enable them to intensify light stress on lower-growing Rosaceae and Fabaceae, amplifying the nitrogen-induced productivity response in grasses (Hautier et al., 2009; DeMalach and Kadmon, 2017).

Despite the increase in plant nitrogen concentrations under some treatments (e.g., high-frequency addition; see **Table 2**), the rise in foliar N was often accompanied by a decline in foliar P and a marked increase in N:P ratios, particularly in Rosaceae and Fabaceae under high nitrogen levels (e.g., Fabaceae N:P reached 124.27 at MN and 118.70 at SN; **Table 2**). According to Koerselman and Meuleman (1996), N:P ratios > 16 indicate phosphorus limitation, while ratios< 14 indicate nitrogen limitation. In our study, the N:P ratios of Rosaceae and Fabaceae frequently exceeded 16 under high-level treatments, suggesting that nitrogen addition induced progressive phosphorus limitation in these functional groups. This is consistent with the global meta-analysis of Vitousek et al. (2010), which showed that terrestrial ecosystems often shift from N to P limitation when N inputs increase, because P is derived primarily from rock weathering and cannot be replenished quickly. Furthermore, a global meta-analysis by Yuan and Chen (2015) demonstrated that N fertilization increases foliar N:P ratios, whereas P fertilization decreases them, indicating a decoupling of N and P cycles under global change. Our results support this decoupling: nitrogen addition alone enhanced N:P ratios in non-grass species, and this P limitation likely constrained their ANPP.

The contrasting responses of Fabaceae to low-frequency versus high-frequency nitrogen addition warrant further ecological explanation. Under low-frequency addition, the single large nitrogen pulse resulted in a rapid surge in soil nitrogen availability, which may have suppressed symbiotic nitrogen-fixation activity. Biological nitrogen fixation is energetically costly, and leguminous plants typically downregulate nodulation and nitrogenase activity when soil nitrogen is abundant (Streeter and Wong, 1988; Van Kessel and Hartley, 2000). This suppression reduces the competitive advantage of Fabaceae over co-occurring species that rely solely on soil nitrogen uptake, leading to decreased biomass accumulation (e.g., Fabaceae ANPP dropped from 28.4 g·m^−2^ at CK to 8.44 g·m^−2^ at LN under low-frequency addition; see **Table 2**). In contrast, high-frequency addition, with multiple smaller applications, may have provided a more gradual and sustained nitrogen supply, allowing Fabaceae to maintain a balance between soil nitrogen uptake and symbiotic fixation (Li et al., 2021). Additionally, the rapid growth of Poaceae under nitrogen addition intensified competition for light and other resources, further disadvantaging Fabaceae under low-frequency regimes (DeMalach and Kadmon, 2017).

Plant nutrient content reflects root acquisition efficiency and internal nutrient allocation, serving as a key indicator of plant growth status (Mao et al., 2020). Two-way ANOVA indicated that plant TN changed significantly in all three functional groups (p< 0.05), while TP showed significant responses in Rosaceae and significant interactions in Fabaceae, but not in Poaceae (see **Table 3**). Plant TC remained largely unaffected. This aligns with the fundamental role of carbon as a structural constituent and the lesser influence of nitrogen addition on carbon assimilation. Under high-frequency addition, plant TN increased significantly across all functional groups (see **Table 2**), suggesting that more frequent nitrogen inputs alleviated nitrogen limitation and enhanced foliar nitrogen acquisition (Su et al., 2019). Nitrogen addition also promoted plant phosphorus uptake by altering soil available phosphorus (Long et al., 2016). However, the “dilution effect” observed in Poaceae under low-frequency LN treatment (TN dropped from 21.91 mg/g at CK to 6.24 mg/g at LN while biomass increased) indicates that rapid biomass accumulation can dilute tissue nutrient concentrations, masking underlying nutrient limitations. It is also worth noting that under low-frequency addition, plant TN in CK was often higher than in the N-addition treatments. This likely resulted from rapid losses of added N when applied as a single large pulse, reducing N availability for plants, whereas under high-frequency addition, split applications sustained soil inorganic N supply and increased plant TN consistently.

The frequency-specific SEMs confirmed that plant nutrient traits were more direct predictors of ANPP than soil properties, although the specific pathways varied between frequencies (**Figure 6**). For Poaceae under high-frequency addition, plant C:N mediated the effect of nitrogen level on ANPP, and the direct Level-ANPP path was also significant. In contrast, under low-frequency addition, no significant mediation through plant nutrients was detected, and the direct effect was not significant (p = 0.060). For Rosaceae, plant TN, TP, C:P and N:P consistently emerged as significant predictors of ANPP across both frequencies. The negative effects of plant TP and C:P on ANPP reinforce the interpretation that nitrogen-induced phosphorus limitation constrains Rosaceae productivity regardless of application frequency, while the involvement of plant TN and N:P further indicates that nitrogen-phosphorus imbalance is a key mechanism underlying the reduced performance of this functional group under nitrogen enrichment.

### 4.3 Differential responses of plant functional groups to nitrogen addition

The divergent responses of plant functional groups to nitrogen addition observed in this study have important ecological implications for alpine grasslands. These functional group-specific responses essentially reflect interspecific differences in nutrient competitive capacity and physiological traits (Bai et al., 2010; Stevens et al., 2006). Under nitrogen enrichment, fast-growing grasses (Poaceae) with rapid growth rates and tall stature can pre-empt light more effectively than slower-growing forbs and legumes, thereby intensifying light competition and suppressing the performance of subordinate functional groups (Hautier et al., 2009; DeMalach and Kadmon, 2017). This shift in competitive hierarchy is a well-documented mechanism driving biodiversity loss after eutrophication (Hautier et al., 2009; Clark and Tilman, 2008). In the long term, such asymmetric competition can alter community composition, favor nitrophilic species at the expense of N-sensitive species, and reduce plant diversity (Stevens et al., 2004; Bai et al., 2010).

Beyond aboveground competition, emerging evidence suggests that functional group-specific responses are also mediated by belowground processes, particularly through differential enzyme activities and microbial interactions. For instance, Dias et al. (2011) demonstrated that plant functional groups in Mediterranean maquis exhibit distinct patterns of nitrate reductase activity, with evergreen sclerophylls showing higher enzyme activity than summer semi-deciduous species, reflecting divergent nitrogen acquisition strategies. This functional differentiation in nitrogen metabolism may influence how different groups respond to nitrogen enrichment. Moreover, Jiao et al. (2025) found that plant functional groups exert stronger effects on soil extracellular enzyme activities and microbial necromass carbon accumulation than planting density, with legumes significantly enhancing soil multifunctionality and nitrogen mineralization rates through rhizosphere microbial stimulation. Similarly, Shen et al. (2025) showed that the co-localization of amino-N and leucine aminopeptidase activity varies among root compartments, indicating that root-microbe interactions for organic nitrogen acquisition are functionally specialized. These studies collectively suggest that nitrogen addition may alter soil enzyme activities and microbial community composition in a functional group-specific manner, which in turn affects nutrient cycling and plant-microbe competition for nitrogen. In alpine grasslands, where environmental conditions are harsh and ecosystem recovery is slow, these nitrogen-induced shifts in both aboveground competitive interactions and belowground microbial processes may have lasting consequences for community succession and ecosystem stability (Tilman, 1999; Isbell et al., 2011). Therefore, understanding how different nitrogen addition regimes (both level and frequency) modulate interspecific competition among plant functional groups—as well as their associated microbial and enzymatic processes, which is critical for predicting future community trajectories, maintaining biodiversity, and sustaining ecosystem functioning under continuous nitrogen deposition.

Consistent with this theory, our study revealed that Poaceae aboveground productivity increased with nitrogen addition level and showed a significant main effect of level (p = 0.009; see **Table 3**), supporting their competitive advantages in growth rate and light acquisition (Bai et al., 2010; Stevens et al., 2006). In contrast, Rosaceae and Fabaceae showed no significant ANPP response to either level or frequency, suggesting that these functional groups are less responsive to short-term nitrogen enrichment, possibly due to their conservative resource-use strategies or reliance on symbiotic nitrogen fixation (Streeter and Wong, 1988; Van Kessel and Hartley, 2000). The frequency-specific SEMs provided further insights into these differential responses (**Figure 6**). For Poaceae, a significant direct positive effect of nitrogen level on ANPP emerged under high-frequency addition, with an additional indirect pathway through plant C:N. Under low-frequency addition, neither the direct path nor any significant mediation was detected. This suggests that split applications during the active growing season enhance nitrogen uptake efficiency and facilitate both direct and indirect growth responses in grasses. For Rosaceae, nitrogen level did not directly affect ANPP under either frequency; instead, its effect was consistently mediated through plant TN, TP, C:P and N:P, with TP and C:P exerting negative effects, this confirming that phosphorus limitation is a robust constraint on Rosaceae productivity regardless of application timing, while the involvement of TN and N:P further highlights the role of N-P stoichiometric imbalance in regulating this response. For Fabaceae, neither frequency exhibited a significant pathway from nitrogen level to ANPP, implying that Fabaceae ANPP is largely insensitive to nitrogen enrichment, likely due to the downregulation of symbiotic nitrogen fixation, which diminishes their competitive advantage relative to grasses. These findings highlight that the relative importance of direct versus indirect pathways depends on both functional group identity and nitrogen addition frequency, underscoring the need for group-specific and frequency-specific considerations when predicting ecosystem responses to ongoing nitrogen deposition.

The inability of Fabaceae to increase ANPP under nitrogen addition, despite elevated TN concentrations under high-frequency SN treatment (30.16 mg/g; see **Table 2**), can be attributed to the suppression of symbiotic nitrogen fixation when soil nitrogen is abundant (Streeter and Wong, 1988; Van Kessel and Hartley, 2000). Biological nitrogen fixation is energetically costly, and leguminous plants typically downregulate nodulation and nitrogenase activity under high soil nitrogen availability, thereby losing their competitive advantage over non-fixing species (Yang et al., 2011). Moreover, the rapid growth of Poaceae under nitrogen addition intensified competition for light, further disadvantaging Rosaceae and Fabaceae (Hautier et al., 2009; DeMalach and Kadmon, 2017). The contrasting responses of Fabaceae to low-frequency versus high-frequency addition (low-frequency addition caused a marked reduction in ANPP at the LN level, while high-frequency addition maintained moderate ANPP) may reflect the ability of more frequent, smaller N pulses to allow finer regulation of nitrogen uptake and better maintenance of symbiotic fixation (Li et al., 2021).

Several limitations of this study should be acknowledged. First, the nitrogen addition experiment was conducted for only one growing season (2021). While this short-term approach allows for the detection of initial responses to nitrogen addition, it cannot capture the long-term dynamics of soil properties, plant community composition, and ecosystem processes that may emerge over extended periods of nitrogen enrichment (Bobbink et al., 2010; Clark and Tilman, 2008). Long-term experiments have shown that the effects of nitrogen deposition on grassland ecosystems can accumulate over time, with delayed responses such as soil acidification, shifts in species composition, and biodiversity loss becoming more pronounced after several years (Stevens et al., 2004; Lu et al., 2014). Therefore, the findings presented here represent the short-term responses of alpine grassland to nitrogen addition, and caution should be exercised when extrapolating these results to longer timescales. Future studies with extended experimental durations are needed to fully elucidate the long-term ecological consequences of nitrogen deposition in this region.

It should be noted that the present study was based on data collected from a single growing season (2021). A more comprehensive understanding of the effects of nitrogen addition on soil properties and plant productivity would be achieved by integrating measurements from multiple stages within the growing season across several years. We are currently accumulating multi-year data in the same study area, which will enable us to provide deeper and more detailed explanations of the observed patterns in the future.

## 5 Conclusions

Increased nitrogen deposition can enhance soil nitrogen availability and stimulate grassland productivity, but excessive nitrogen inputs may also disrupt plant nutrient balance and alter species interactions. In this study, we found that nitrogen addition level, rather than addition frequency, was the primary driver of ANPP in the Bayanbulak alpine grassland. The positive effect of nitrogen level on ANPP was largely attributable to increased soil nitrate and available phosphorus, which alleviated nitrogen limitation in grasses. In contrast, addition frequency played only a secondary, indirect role by modulating soil-plant nutrient coupling without independently affecting productivity. Among functional groups, Poaceae exhibited a strong and direct ANPP response to nitrogen level under high-frequency addition, benefiting from their competitive advantages in light acquisition and nitrogen use efficiency. Rosaceae and Fabaceae, however, showed no significant ANPP response; instead, they experienced progressive phosphorus limitation, as evidenced by elevated N:P ratios under high nitrogen treatments. Fabaceae displayed contrasting responses to nitrogen frequency, with low-frequency pulses suppressing growth via inhibition of symbiotic nitrogen fixation, while high-frequency additions allowed better maintenance of productivity. These findings demonstrate that nitrogen deposition alters grassland productivity in a functional-group-specific manner, primarily through nitrogen level effects, and that the resulting nutrient imbalances may reshape community composition. To predict and mitigate the ecological consequences of ongoing nitrogen deposition, future management and policy efforts should consider not only the total nitrogen load but also the differential sensitivities of plant functional groups, particularly the risk of phosphorus limitation in non-grass species.

## Data Availability

The raw data supporting the conclusions of this article will be made available by the authors, without undue reservation.
