# Supplementary material for: Nitrogen addition level is more influential to aboveground productivity than nitrogen addition frequency in an alpine grassland
Source: Front Plant Sci. 2026 Aug 14;17:1821183. doi: 10.3389/fpls.2026.1821183 (PMC13522212; doi:10.3389/fpls.2026.1821183)
Supplement: Supplementary file 1 [file DataSheet1.pdf]

Nitrogen Addition Level is More Influential to Aboveground Productivity Changes than Nitrogen Addition Frequency

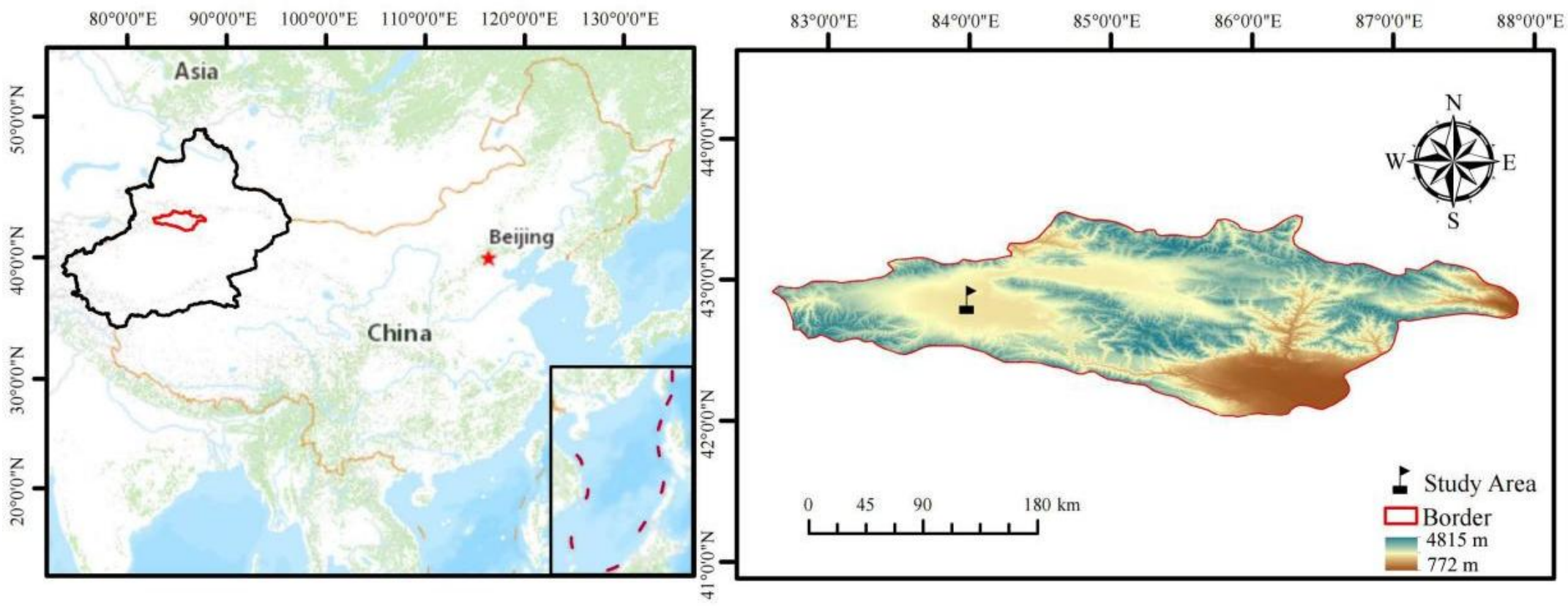

Figure.S1 Overview of the study area

SN	LN	HN	MN	LN	CK	SN	MN
MN	CK	SN	LN	CK	MN	HN	LN
SN	HN	LN	CK	HN	LN	SN	CK
CK	LN	MN	HN	SN	CK	LN	HN
MN	HN	SN	CK	MN	HN	MN	SN

Figure.S2 Quadrat distribution

Note: Control Check (CK) (0 g·m^{-2}); Low N (LN) (5 g·m^{-2}); Medium N (MN) (10 g·m^{-2}); High N (HN) (15 g·m^{-2}); Severe N (SN) (20 g·m^{-2}). Left: low-frequency addition (single application); right: high-frequency addition (three split applications)

Table S1 Measurement methods

**Measure indicators**		**Measure methods**
**Plant nutrient content**	Plant TC	$K_2Cr_2O_7$-$FeSO_4$ external heating method
	Plant TN	Kjeldahl method
	Plant TP	$HClO_4$-H_2SO_4 molybdenum antimony colorimetric method
**Soil physicochemical properties**	Soil pH	pH meter method
	SSC	Potentiometric method
	SWC	Drying-out method
	Soil TOC	$K_2Cr_2O_7$-$FeSO_4$ external heating method
	Soil TN	Kjeldahl method
	Soil AN	Indophenol blue colorimetric method
	Soil NN	Ultraviolet spectrophotometry
	Soil TP	$HClO_4$-H_2SO_4 molybdenum antimony colorimetric method
	Soil AP	$NaHCO_3$ leaching-molybdenum antimony colorimetric method

Table S2 Effects of nitrogen addition level and frequency on community aboveground productivity

	Frequency	Level	Community Aboveground productivity(g/m^2)
Community	low-frequency	CK	131.38±9.21a
		LN	139.03±12.62a
		MN	146.57±4.18a
		HN	178.82±18.86a
		SN	178.82±18.86a
	high-frequency	CK	123.79±8.13a
		LN	121.55±9.94a
		MN	172.30±16.19b
		HN	162.42±4.19ab
		SN	187.77±3.55b

Note: Different lowercase letters within the same frequency (Low or High) indicate significant differences among nitrogen addition levels (CK, LN, MN, HN, SN) at $p < 0.05$. Levels sharing the same letter are not significantly different.

Table S3 Effects of nitrogen addition level and frequency on community aboveground productivity

		Community Aboveground productivity	
Functional groups	N addition	F	P
Community	level	10.930	< 0.001
	frequency	0.073	0.789
	level*frequency	1.448	0.243

Table S4 One-way ANOVA results testing the individual effects of nitrogen addition level and addition frequency on soil physicochemical properties

Soil Layer	N Addition	Statistic	Soil AN	Soil AP	C:N	C:P	N:P	Soil NN	SSC	SWC	Soil TN	Soil TOC	Soil TP	Soil pH
0-10 cm	Level	F-value	0.979	2.250	0.668	0.558	0.323	0.895	0.259	0.418	0.652	1.560	0.700	0.424
		P-value	0.431	0.084	0.619	0.694	0.861	0.477	0.902	0.794	0.629	0.207	0.597	0.790
	Frequency	F-value	0.224	1.810	0.042	0.198	0.000	16.200	2.880	0.121	0.043	3.090	0.013	0.070
		P-value	0.639	0.187	0.838	0.659	0.996	**<0.000**	0.098	0.730	0.836	0.087	0.911	0.793
10-20 cm	Level	F-value	0.575	0.956	1.630	0.871	0.920	4.100	0.888	1.030	0.157	1.830	1.560	2.310
		P-value	0.683	0.444	0.188	0.491	0.464	**0.008**	0.481	0.408	0.958	0.144	0.207	0.077
	Frequency	F-value	0.344	0.848	0.107	0.470	0.121	4.980	2.920	3.300	0.062	5.460	0.008	0.031
		P-value	0.561	0.363	0.746	0.497	0.730	**0.032**	0.096	0.077	0.805	**0.025**	0.929	0.862

Table S5 Two-way ANOVA results testing the effects of nitrogen addition level and frequency on soil physicochemical properties

**Soil physicochemical properties**		**SWC**		**Soil pH**		**SSC**		**Soil TOC**		**Soil TN**		**Soil AN**		**Soil NN**		**Soil TP**		**Soil AP**		**C:N**		**C:P**		**N:P**	
**Soil layers**	**N addition**	**F**	**P**	**F**	**P**	**F**	**P**	**F**	**P**	**F**	**P**	**F**	**P**	**F**	**P**	**F**	**P**	**F**	**P**	**F**	**P**	**F**	**P**	**F**	**P**
	**level**	0.367	0.83	0.382	0.820	0.391	0.813	1.724	0.171	0.615	0.655	1.089	0.380	1.788	0.157	0.625	0.648	2.700	**0.049**	0.674	0.615	0.511	0.728	0.316	0.865
**0-10**	**frequency**	0.102	0.752	0.061	0.807	3.840	0.059	3.204	0.084	0.040	0.842	0.255	0.617	22.991	**<0.000**	0.013	0.911	2.402	0.132	0.042	0.839	0.177	0.677	<0.000	0.996
	**level*frequency**	0.150	0.962	0.364	0.833	4.758	**0.004**	1.564	0.210	0.740	0.572	2.179	0.095	4.224	**0.008**	0.235	0.917	2.411	0.071	1.315	0.287	0.465	0.761	1.083	0.383
	**level**	0.862	0.498	2.429	0.069	0.945	0.452	2.278	0.084	0.157	0.958	0.563	0.692	7.125	**<0.000**	1.558	0.211	0.905	0.473	1.486	0.231	0.948	0.450	1.023	0.411
**10-20**	**frequency**	2.847	0.102	0.038	0.848	2.925	0.098	6.613	**0.015**	0.058	0.812	0.327	0.571	10.351	**0.003**	0.009	0.927	0.803	0.377	0.106	0.747	0.512	0.480	0.136	0.714
	**level*frequency**	0.215	0.928	1.691	0.178	1.076	0.386	1.718	0.172	1.201	0.331	0.981	0.433	5.105	**0.003**	1.249	0.312	0.587	0.675	0.443	0.777	1.900	0.136	2.199	0.093
